# PPARγ agonist alleviates calcium oxalate nephrolithiasis by regulating mitochondrial dynamics in renal tubular epithelial cell

**DOI:** 10.1371/journal.pone.0310947

**Published:** 2024-09-26

**Authors:** Junfa Liu, Xingyang Liu, Lizhe Guo, Xiongfei Liu, Qian Gao, E. Wang, Zhitao Dong

**Affiliations:** 1 Department of Urology, The Second Xiangya Hospital, Central South University, Changsha, China; 2 Department of Anesthesiology, Xiangya Hospital Central South University, Changsha, China; 3 Center for Translational Medicine and Jiangsu Key Laboratory of Molecular Medicine, Medical School of Nanjing University, Nanjing, China; China Medical University, TAIWAN

## Abstract

**Background:**

Kidney stone formation is a common disease that causes a significant threat to human health. The crystallization mechanism of calcium oxalate, the most common type of kidney stone, has been extensively researched, yet the damaging effects and mechanisms of calcium oxalate crystals on renal tubular epithelial cells remain incompletely elucidated. Regulated mitochondrial dynamics is essential for eukaryotic cells, but its role in the occurrence and progression of calcium oxalate (CaOx) nephrolithiasis is not yet understood.

**Methods:**

An animal model of calcium oxalate-related nephrolithiasis was established in adult male Sprague‒Dawley (SD) rats by continuously administering drinking water containing 1% ethylene glycol for 28 days. The impact of calcium oxalate crystals on mitochondrial dynamics and apoptosis in renal tubular epithelial cells was investigated using HK2 cells *in vitro*. Blood samples and bilateral kidney tissues were collected for histopathological evaluation and processed for tissue injury, inflammation, fibrosis, oxidative stress detection, and mitochondrial dynamics parameter analysis.

**Results:**

Calcium oxalate crystals caused higher levels of mitochondrial fission and apoptosis in renal tubular epithelial cells both in vivo and in vitro. Administration of a PPARγ agonist significantly alleviated mitochondrial fission and apoptosis in renal tubular epithelial cells, and improved renal function, accompanied by reduced levels of oxidative stress, increased antioxidant enzyme expression, alleviation of inflammation, and reduced fibrosis in vivo.

**Conclusion:**

Our results indicated that increased mitochondrial fission in renal tubular epithelial cells is a critical component of kidney injury caused by calcium oxalate stones, leading to the accumulation of reactive oxygen species within the tissue and the subsequent initiation of apoptosis. Regulating mitochondrial dynamics represents a promising approach for calcium oxalate nephrolithiasis.

## 1. Introduction

Kidney stones are a common disease of the urinary system, with increasing prevalence worldwide in both children and adults [1,2], and the incidence of recurrence within a decade exceeds 50% [3]. Previous research has found that patients with kidney stones are at an increased risk of adverse health outcomes, such as end-stage renal disease [4] and cardiovascular disease [5]. Calcium oxalate (CaOx) nephrolithiasis is the most common type of kidney stone, accounting for approximately 70% of cases [2]. It has been proven that the primary factor contributing to the formation of kidney stones is increased urinary calcium excretion, which may result from increased intestinal absorption, enhanced bone resorption, or decreased renal reabsorption of calcium [6]. However, the damaging effects and mechanisms of calcium oxalate crystals on renal tubular epithelial cells remain incompletely elucidated. Moreover, the current treatment options still have certain limitations.

The formation of kidney stones is a progressive pathological process, and even in kidney of patients that present with symptoms of urinary obstruction, calcium oxalate crystals in their early crystalline phase would also be existed in the renal interstitium. Numerous studies have shown that calcium oxalate crystals in the renal interstitium can directly influence the biological behavior of renal tubular epithelial cells through direct contact causing oxidative stress, inflammation, and other direct damage [7–9]. Clinical studies have confirmed that crystalline nephropathy is often associated with a worse outcome [10]. However, surgical removal of stones or ultrasonic lithotripsy is only effective for urinary tract stones, and currently, there are no effective clinical methods for eliminating calcium oxalate crystals in the renal interstitium.

Many studies have indicated that apoptosis of renal tubular epithelial cells (TECs) plays a central role in stone-related renal function impairment [11,12]. The damage or loss of TECs not only directly reduces renal function but also exacerbates calcium reabsorption dysfunction, thereby accelerating kidney stone formation [6]. Reactive oxygen species (ROS) are common mediators of apoptosis [13]. Mitochondrial dysfunction is considered an important source of oxidative stress and is closely related to the development of kidney stone-associated diseases [14]. Hence, it is of great importance to investigate the role of mitochondrial homeostasis in the occurrence and development of calcium oxalate nephrolithiasis and elucidate its underlying molecular mechanisms.

Mitochondrial quality control is essential for maintaining mitochondrial homeostasis and includes multiple quality control mechanisms, such as antioxidant defense, protein quality control, mitochondrial DNA repair, mitochondrial dynamics, mitophagy and mitochondrial biogenesis [15]. In this study, we confirmed the severe dysfunction of mitochondrial dynamics in CaOx nephrolithiasis, resulting in significantly elevated mitochondrial fission and TECs apoptosis. We sought to elucidate the role of imbalanced mitochondrial dynamics in calcium oxalate nephrolithiasis and the ameliorative effect of PPARγ agonist treatment on mitochondrial damage in TECs.

## 2. Materials and methods

### 2.1 Animals

All experiments were conducted in accordance with the recommendations of national and international animal protection and ethical guidelines and were approved by the Animal Research Ethics Committee of Xiangya Hospital, Central South University (permit code: 202301003). Adult male Sprague‒Dawley (SD) rats weighing 250–300 g were provided by the Laboratory Animal Center of Central South University, China. Animals were housed on a 12:12 hour light-dark cycle under constant temperature (24 ± 1°C) in standard cages and fed a standard diet. All experimental animals were euthanized using high-concentration isoflurane inhalation to minimize their suffering.

### 2.2 Animal model for calcium oxalate (CaOx) nephrolithiasis and treatment

The kidney stone rat model was established as described previously [16]. The control group was given normal drinking water for 28 days. The kidney stone group was given drinking water containing 1% ethylene glycol (EG, Sigma‒Aldrich, Buchs, Switzerland) continuously for 28 days. The PPARγ agonist troglitazone group (TR group) was given daily oral administration of troglitazone (HY-50935, MedChem Express, Monmouth Junction, NJ) at a dose of 3 mg/kg [17] and was also given drinking water containing 1% EG freely for 28 days. After 28 days, blood samples were collected from each group of rats for biochemical indicator detection, and bilateral kidneys were collected for histological examination.

### 2.3 Plasma collection and analysis

On Day 28, blood was collected from all rat groups. Rats were anesthetized with sevoflurane, and blood was collected from the inferior vena cava in a tube coated with heparin and centrifuged for 10 min at 2000 × g (4°C) to isolate plasma. The plasma was stored at -80°C. Urea nitrogen, creatinine, and cystatin C concentrations were measured using an Auto Analyzer (Beckman Coulter, AU5821, CA, USA).

### 2.4 Cell culture and treatment

The human proximal PTC line (HK2) was purchased from Abiowell Biotechnology Co., Ltd. (Changsha, China). The cell culture medium for HK2 cells was DMEM/F12 (Gibco, 11330500). All cells were cultured with 10% fetal bovine serum (HyClone, SV30087.03) and 1% penicillin–streptomycin double antibiotics (Abiowell, AWH0529a, China) at 37°C with 5% CO_2_. HK2 cells were seeded on six-well plates in the absence or presence of calcium oxalate monohydrate (COM). To better study the functional changes in HK2 cells under COM stimulation, the cells were treated with 100, 200, and 300 μg/mL COM and incubated for 48 h. Furthermore, the cells were grouped into control + DMSO, CaOx + DMSO, and CaOx + TR groups. For the CaOx + TR group, cells were treated with 1 μM troglitazone 12 hours before treatment with COM for 48 hours. In the CaOx + DMSO group, cells were treated with an equal volume of DMSO before treatment with COM. In the control + DMSO group, cells were only treated with an equal volume of DMSO at the same time.

### 2.5 Morphologic analysis and histologic scoring

Kidney samples were fixed in 4% paraformaldehyde at room temperature, embedded in paraffin, and cut into 5-μm-thick sections. Subsequently, the sections were separately stained with hematoxylin and eosin (H&E), Sirius Red staining, and periodic acid-Schiff (PAS) stain. Collagen accumulation was further determined by Sirius Red staining in a blinded manner. For Sirius Red staining, red collagen deposition was taken as a positive signal, and the percentage of positive area in the entire visual field was recorded and averaged across the ten fields for each section. PAS and H&E staining strategies can be used to evaluate the degree of damage to renal tubules [18–20]. Kidney injury was assessed by morphometric analysis using the following criteria based on previous research reports [2]: no injury (score 0), tubular dilatation and loss of brush border (score 1), tubular epithelial cell vacuolization (score 2), apical blebbing (score 3), and epithelial cell sloughing and granular casts (score 4). The degree of kidney injury was quantified using the following formula: (number of tubules with a score greater than 0 × corresponding score) divided by the total number of tubules examined. The renal cortex was evaluated for each section by examining 5 high-power fields (40× magnification).

Polarized light optical microphotography (NE 910, Nexcope) was performed to evaluate the deposition of stone crystals in kidney tissue sections. The total number of crystals present was counted blindly in 5 fields per section.

### 2.6 Western blot analysis

Total proteins were extracted from renal tissue in RIPA buffer containing protease inhibitors (Servicebio, Wuhan, China). The protein extracts were separated by 10% SDS‒PAGE and transferred to PVDF membranes. The membranes were then blocked with 5% skim milk in PBS with 0.1% Tween 20 (PBST) at room temperature for 1 hour and incubated overnight at 4°C with primary antibodies specific for Caspase-3 (ab179517, Abcam, 1:1000), Bax (ab32503, Abcam, 1:1000), Bcl-2 (ab196495, Abcam, 1:1000), DRP1 (ab184247, Abcam, 1:1000), OPA1 (67589S, CST, 1:1000), Mitofusin-2 (9482S, CST, 1:1000), and GAPDH (A531, Bioworld, 1:5000). For more antibody details, please refer to **S1 Table** in S1 File. The blots were then probed with appropriate secondary antibodies for 1 h at room temperature. Chemiluminescence reagent (K-12045-D50, Advansta, San Jose, CA) was used to detect the bands. The obtained images were analyzed using ImageJ software.

### 2.7 RNA isolation and qRT‒PCR gene expression

Total ribonucleic acid (RNA) was extracted from renal tissue using a Total RNA Kit II (R6934-01; Omega Biotek, Norcross, GA), and messenger RNA (mRNA) was reverse transcribed into complementary DNA (cDNA) using an RT Reagent Kit with gDNA Eraser (No. RR047A; Takara, Tokyo, Japan) according to the manufacturer’s protocol. Real-time PCR was performed using All-in-One^TM^ qPCR Mix (No: QP001; GeneCopoeia, Germantown, MD) based on standard protocols. GAPDH was used for the normalization of relative gene expression. Primers were designed and synthesized by Sangon Biotech (Shanghai, China), and most primer sequences are listed in **S2 and S3 Tables** in S1 File. Primers for NF-κb were purchased from GeneCopoeia (Germantown, MD).

### 2.8 TUNEL staining for apoptosis

Slides were evaluated using the TUNEL method with an *in situ* cell death detection kit (Roche® Life Science, Shanghai, China). After dewaxing the paraffin sections, proteinase K was used for permeabilization. After incubation with a membrane-breaking working solution at room temperature, the slices were incubated with a mixture of dUTP and TdT at 37°C and finally restained with hematoxylin. Cells were regarded as TUNEL-positive when the nuclei were stained brown. Ten fields were randomly selected, and TUNEL-positive cells were counted in a blinded manner.

### 2.9 Detection of advanced oxidation protein product (AOPP)

The advanced oxidation protein product (AOPP) assay was performed using the AOPP Assay Kit (ab242295, Cambridge, UK) according to the manufacturer’s protocol. The results were then normalized to the protein concentrations of each test.

### 2.10 Immunofluorescent staining and quantification of mitochondrial morphology

Direct immunofluorescent staining of GRP75 was performed to label mitochondria in renal tissue sections. GRP75 (75-kDa glucose-regulated protein) is a major component of the mitochondria-associated membrane [21]. GRP75 is stably expressed in mitochondria, and mitochondria from cells stained with anti-GRP75 antibodies can display punctate and tubular morphology [22]. After deparaffinization of the paraffin sections, antigen retrieval was performed. The tissue sections were incubated with 3% H_2_O_2_ for 10 minutes to remove endogenous peroxidase. The sections were then placed in phosphate-buffered saline (PBS) (pH 7.4) and washed 3 times for 5 min each. Then, the sections were blocked with 3% BSA (containing 0.3% Triton-X 100) at 37°C for 90 minutes. Primary antibody against GRP75 (anti-GRP75/MOT, ab2799, Abcam, 1:50) was added to the sections and incubated at 4°C overnight. The sections were then incubated in Alexa Fluor 488 (ab150113, Abcam, 1:500) and counterstained with DAPI. Finally, the sections were imaged using a Zeiss Apotome.2 microscope. According to the protocol provided by previous research [23], we used Fiji/ImageJ software to analyze the morphological features of mitochondria, including the mitochondrial count, average area, average perimeter, and aspect ratio, as auxiliary evidence for well-characterized proteins involved in mitochondrial fission and fusion.

The cells were stained with 100 nM MitoTracker Red (Invitrogen, M7512) for 30 minutes. After that, they were fixed for 10 minutes with 4% paraformaldehyde and permeabilized with 0.1% Triton X-100 for 10 minutes. For OPA1 staining, the cells were incubated overnight at 4°C with a specific primary antibody (67589S, CST, 1:800) in 1% BSA, followed by incubation with a secondary Alexa Fluor 488 antibody (ab150113, Abcam, 1:500) at room temperature for 1 hour. DAPI was used to stain DNA. The images were captured using a Zeiss Apotome.2. Consistent with the previous description, the area, perimeter, aspect ratio, and form factor of the mitochondria were quantified using Fiji/ImageJ software.

### 2.11 Detection of HK2 apoptosis and necrosis

Cell apoptosis and necrosis were determined using an Apoptosis and Necrosis Assay Kit (Beyotime Biotechnology, China). According to the manufacturer’s instructions, after washing the cells once with PBS, staining buffer, Hoechst 33342, and propidium iodide (PI) were added sequentially. The mixture was gently mixed and kept on ice in the dark for 20 minutes. After another wash with PBS, the cells were observed under an inverted fluorescence microscope (N2Ti2-A, Nikon). The proportions of apoptotic and necrotic cells were analyzed using ImageJ software.

### 2.12 Measurement of ROS

Cell‐permeable H2DCF (HY-D0940, MCE) was applied to measure the intracellular total ROS levels. HK2 cells were cultured in a 35 mm glass-bottomed culture dish. After washing with PBS three times, HK2 cells were treated with 5 μM H2DCF for 30 minutes at 37°C in the dark. The images were visualized on an Axio Observer 7 microscope (Zeiss).

### 2.13 Statistical analysis

The significance between two groups was assessed using the two-tailed Student’s t test. For pairwise comparisons among more than two groups, the significance was evaluated using one-way analysis of variance with a post hoc Dunnett’s–Bonferroni post test. All data are expressed as the mean ± standard error of the mean (SEM). The significance level was defined as *P* < 0.05. Statistical analyses were performed using GraphPad Prism 9 software (GraphPad Software Inc., USA).

## 3. Results

### 3.1 Calcium oxalate nephrolithiasis exhibited elevated oxidative stress and TEC apoptosis in the kidney

**Fig 1:**
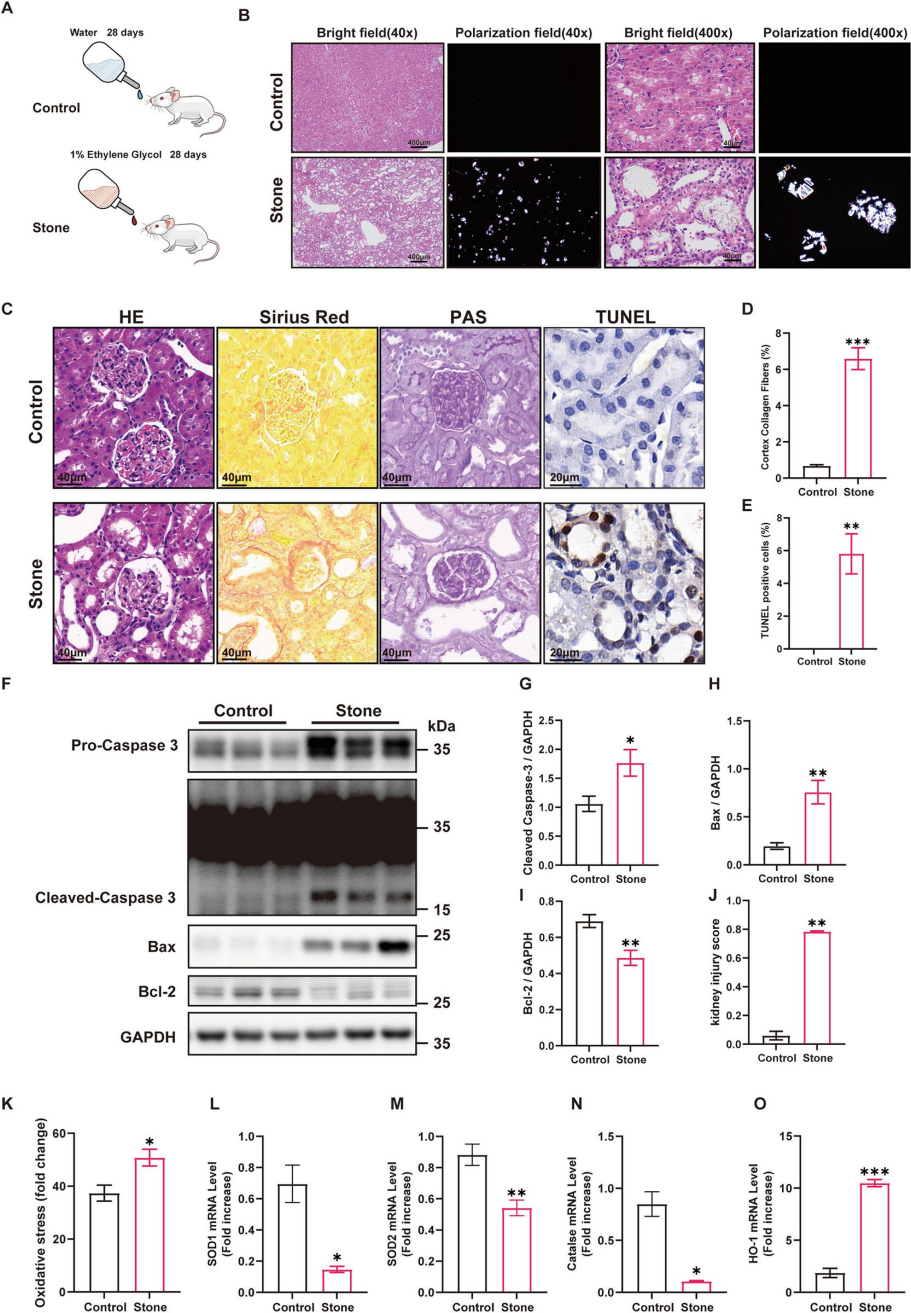
The calcium oxalate nephrolithiasis induced by EG exhibits significant crystal deposition, fibrosis, apoptosis, and increased oxidative stress levels in rats. **(A)** Experimental flowchart for the EG-induced calcium oxalate nephrolithiasis model; **(B)** Polarized light optical microscopy showed large crystals in the kidneys of rats in the stone group; **(C)** Paraffin-embedded sections of renal tissue were stained with H&E, Sirius Red staining, PAS staining, and TUNEL staining; **(D)** The degree of interstitial fibrosis in the renal cortex, n = 30 fields per group; **(E)** The average number of TUNEL-positive cells per high-power field, n = 15 fields per group; **(F)** Lysates from renal tissue were analyzed by western blotting with the indicated antibodies; **(G-I)** The relative protein quantification of F, n = 5 rats per group; **(J)** Kidney injury was scored in different groups, n = 15 fields per group; **(K)** Oxidative stress levels in renal tissue were measured by the AOPP test; **(L-O)** Gene expression levels of different renal tissues were determined by RT‒qPCR. Data are represented as the mean±SEM; * represents *P*<0.05, ** represents *P*<0.01, *** represents *P*<0.001.

We established a rat model of calcium oxalate nephrolithiasis by adding 1% EG to the drinking water for 28 days (Fig 1A). Through polarized light optical microphotography, a substantial deposition of calcium oxalate crystals in renal tubules was clearly observed in the pathological sections of kidneys in the stone group, the calcium oxalate crystals were primarily located within the renal tubules of both the renal cortex and medulla. (Fig 1B). H&E and PAS staining revealed abnormalities in renal histologic structure, such as glomerular atrophy, tubular epithelial cell swelling, vacuolar degeneration, severe tubular dilatation, and shedding of small numbers of cilia and epithelial cells (Fig 1C). Sirius Red staining further revealed interstitial fibrosis in rats in the stone group (Fig 1C and 1D). Apoptosis in the kidney was detected through a TUNEL assay, which indicated that a greater rate of apoptosis was found in TECs of the stone group compared to the control group (Fig 1C and 1E). Consistent with previous findings, western blot analysis indicated that apoptosis was significantly activated in the kidneys of rats in the stone group (Fig 1F–1J). Oxidative stress is a common mediator of apoptosis [13]; hence, we detected advanced oxidation protein products (AOPP) in homogenized kidney tissue samples to confirm the level of oxidative stress in kidneys from both groups and found significantly elevated levels of oxidative stress in the kidneys of the stone group (Fig 1K). In addition to the compensatory upregulation of HO-1, the mRNA levels of key antioxidant enzymes (SOD1, SOD2, CAT) in the kidneys of rats in the stone group were all decreased (Fig 1L–1O). Taken together, these observations indicate that increased oxidative stress might be a crucial factor in the induction of apoptosis in TECs by calcium oxalate.

### 3.2 Elevated mitochondrial fission and decreased mitochondrial biogenesis were observed in calcium oxalate nephrolithiasis

**Fig 2:**
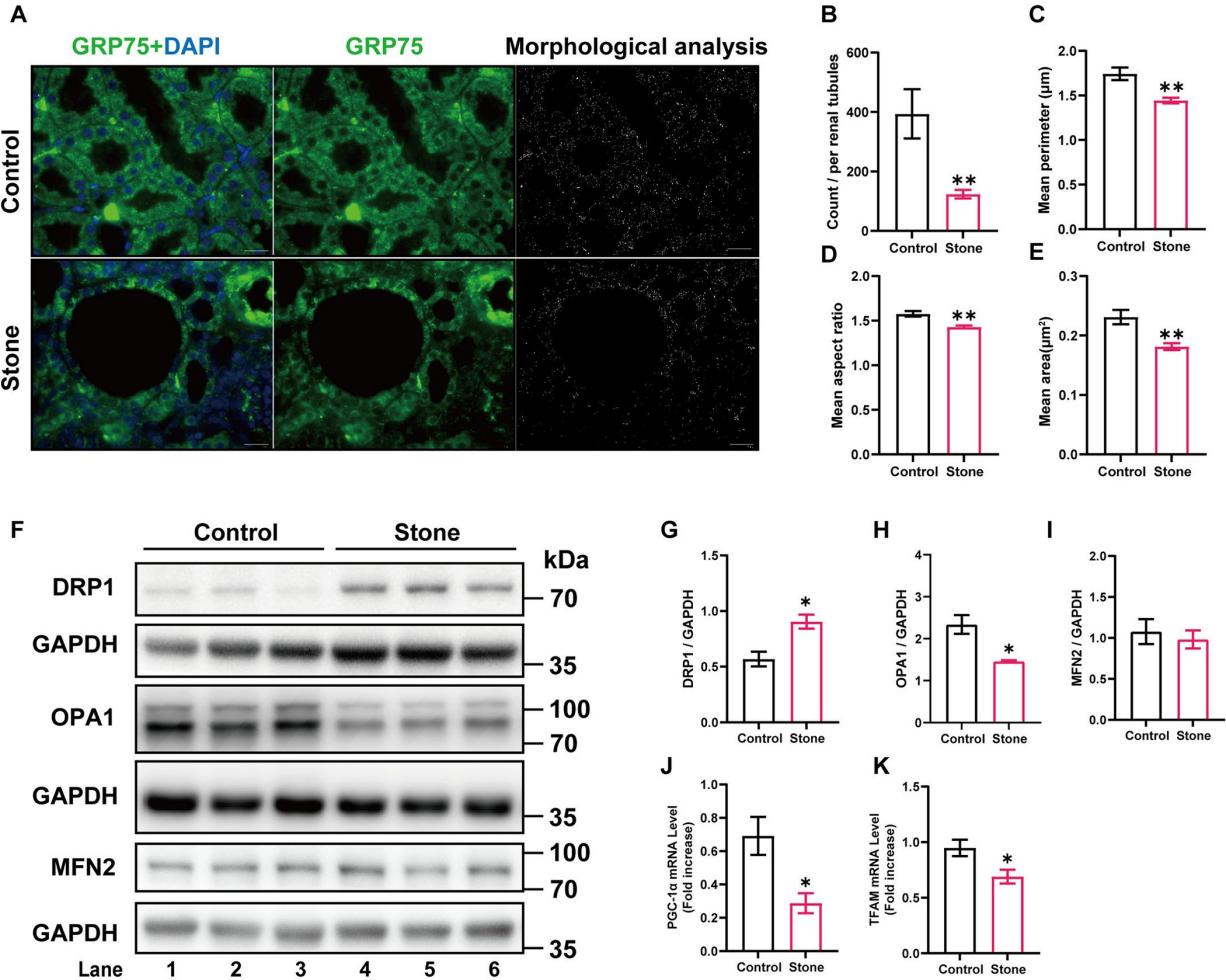
The rat calcium oxalate nephrolithiasis model exhibits significant mitochondrial dynamics disorder and damage to biosynthesis. **(A)** Immunofluorescence staining of the mitochondrial protein GRP75 and schematic representation of mitochondrial morphology in the renal cortex regions of different groups. Scale bar = 20 μm; **(B-E)** The mean count of mitochondria per renal tubule, mean area (um^2^), mean perimeter (um), and mean aspect ratio of mitochondria in the different groups were quantified, n = 15 renal tubules per group; **(F)** Lysates from renal tissue were analyzed by western blotting with the indicated antibodies; **(G-I)** The relative protein quantification of F, n = 5 rats per group; **(J-K)** Gene expression levels of different renal tissues were determined by RT‒qPCR, n = 5 rats per group. Data are represented as the mean ± SEM; * represents *P*<0.05, ** represents *P*<0.01, *** represents *P*<0.001.

Mitochondria not only serve as a main source of ROS, but under physiological conditions, the intracellular levels of ROS also depend on the maintenance of the normal mitochondrial antioxidant defense system at lower levels [15]. Immunofluorescence staining of the mitochondrial protein GRP75 revealed that compared with those of the control group, the count, mean area, and perimeter of the mitochondria in renal tubules of rats in the stone group were significantly reduced, and a decreased mean aspect ratio also indicated increases in mitochondrial fission and fragmentation of the mitochondria in renal tubules of rats in the stone group (Fig 2A–2E). Consistent with previous findings, western blot analysis indicated that the expression level of DRP1, which is responsible for mitochondrial fission, was increased in the stone group, while the expression levels of the mitochondrial fusion protein OPA1 were decreased (Fig 2F–2I). In addition, the mRNA levels of PGC1-α and TFAM were decreased in the stone group (Fig 2J–2K), indicating a disruption in mitochondrial biogenesis. Taken together, these observations indicated the existence of poor mitochondrial quality control in calcium oxalate nephrolithiasis.

### 3.3 Calcium oxalate induced elevated mitochondrial fission and apoptosis in HK2 cells

**Fig 3:**
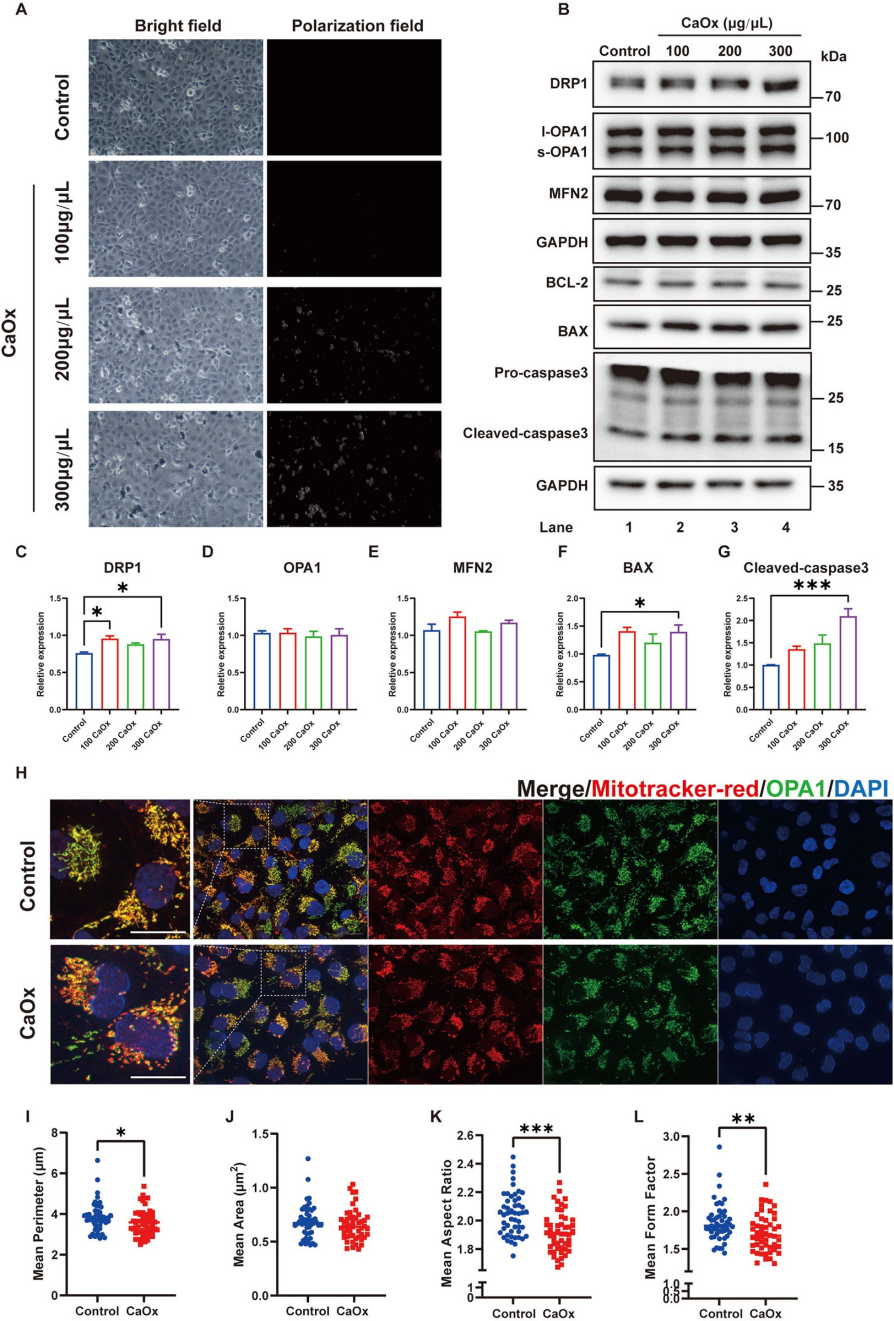
Treatment with COM induced mitochondrial fission in HK2 cells. **(A)** Bright-field and polarized light images of HK2 cells treated with different concentrations of COM (10×). **(B)** western blotting of HK2 cells with the indicated antibodies following treatment with different concentrations of COM for 48 h. **(C-G)** The relative protein quantification of F, n = 3 per group. **(H)** HK2 cells were treated with 300 μg/ml COM as indicated and stained with MitoTracker Red and GRP75 to visualize mitochondrial morphology. The left panel is an enlarged view of the boxed region in the right panel. Scale bar = 20 μm; **(I-L)** The means of perimeter (μm), area (μm^2^), aspect ratio, and form factor of mitochondria in different groups were quantified, n = 50 cells per group. Data are represented as the mean ± SEM; * represents *P*<0.05, ** represents *P*<0.01, *** represents *P*<0.001.

To investigate calcium oxalate damage in TECs, we treated HK2 cells with calcium oxalate monohydrate (COM) (Fig 3A) and found that treatment with COM for 48 hours significantly induced apoptosis activation in HK2 cells, with an increase in DRP1 expression, consistent with the *in vivo* experimental results (Fig 3B–3G). Based on the results of the concentration gradient experiments, we chose 300 μg/ml as the treatment concentration for the subsequent experiments, and we analyzed the mitochondrial morphology of COM-treated HK2 cells via immunofluorescence and stained for mitochondria-specific markers (mitochondrial protein OPA1 and MitoTracker Red). The mitochondrial network was found to be more fragmented in the CaOx group (Fig 3H). The mean mitochondrial area was significantly decreased in the CaOx group, while the mean mitochondrial perimeter remained unaffected (Fig 3I and 3J), and the decreases in the mean aspect ratio and mean form factor suggested that the mitochondria in the CaOx group were more spherical in shape, indicating increased mitochondrial fission in the CaOx group (Fig 3K and 3L). These results indicate that calcium oxalate crystals might promote oxidative stress accumulation and apoptosis in TECs by inducing abnormally increased mitochondrial fission.

### 3.4 Treatment with a PPARγ agonist rescued imbalanced mitochondrial dynamic and apoptosis in COM-treated HK2 cells

**Fig 4:**
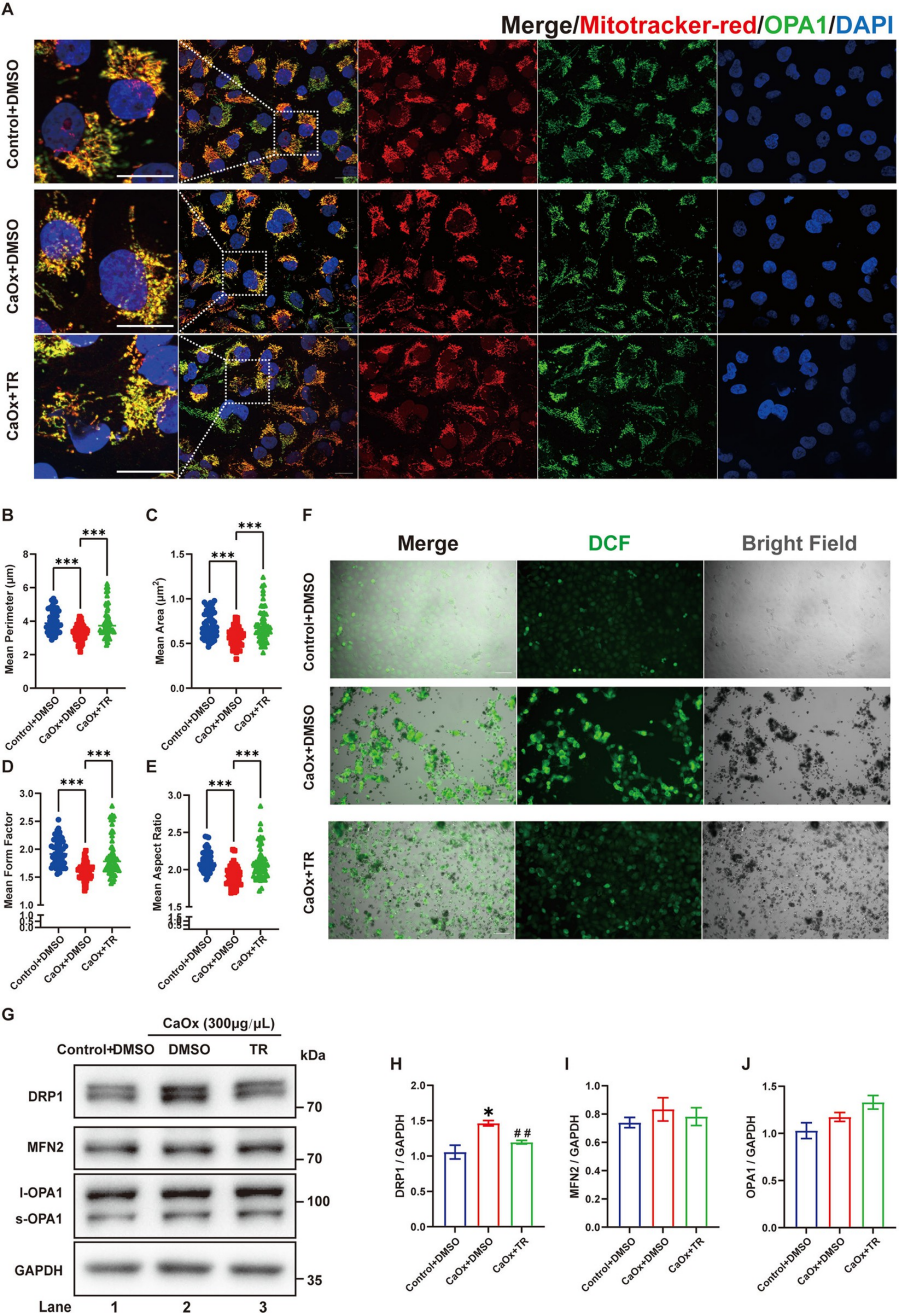
Treatment with a PPARγ agonist improved COM-induced mitochondrial fragmentation and ROS levels in HK2 cells. **(A)** HK2 cells were treated with COM or COM and troglitazone as indicated and stained with MitoTracker Red and GRP75 to visualize mitochondrial morphology. The left panel is an enlarged view of the boxed region in the right panel. Scale bar = 20 μm; **(B-E)** The means of perimeter (μm), area (μm^2^), form factor, and aspect ratio of mitochondria in different groups were quantified, n = 50 cells per group; **(F)** The total ROS levels were analyzed by H2DCF fluorescence. Scale bar = 20 μm. **(G)** Lysates from HK2 cells were analyzed by western blotting with the indicated antibodies. **(H-J)** The relative protein quantification of G, n = 3 per group. Data are represented as the mean ± SEM; * *P*<0.05, ** *P*<0.01, *** *P*<0.001 *vs*. the control + DMSO group; ^#^ *P*< 0.05, ^##^ *P*< 0.01, ^###^ *P*< 0.001 *vs*. the CaOx + DMSO group.

**Fig 5:**
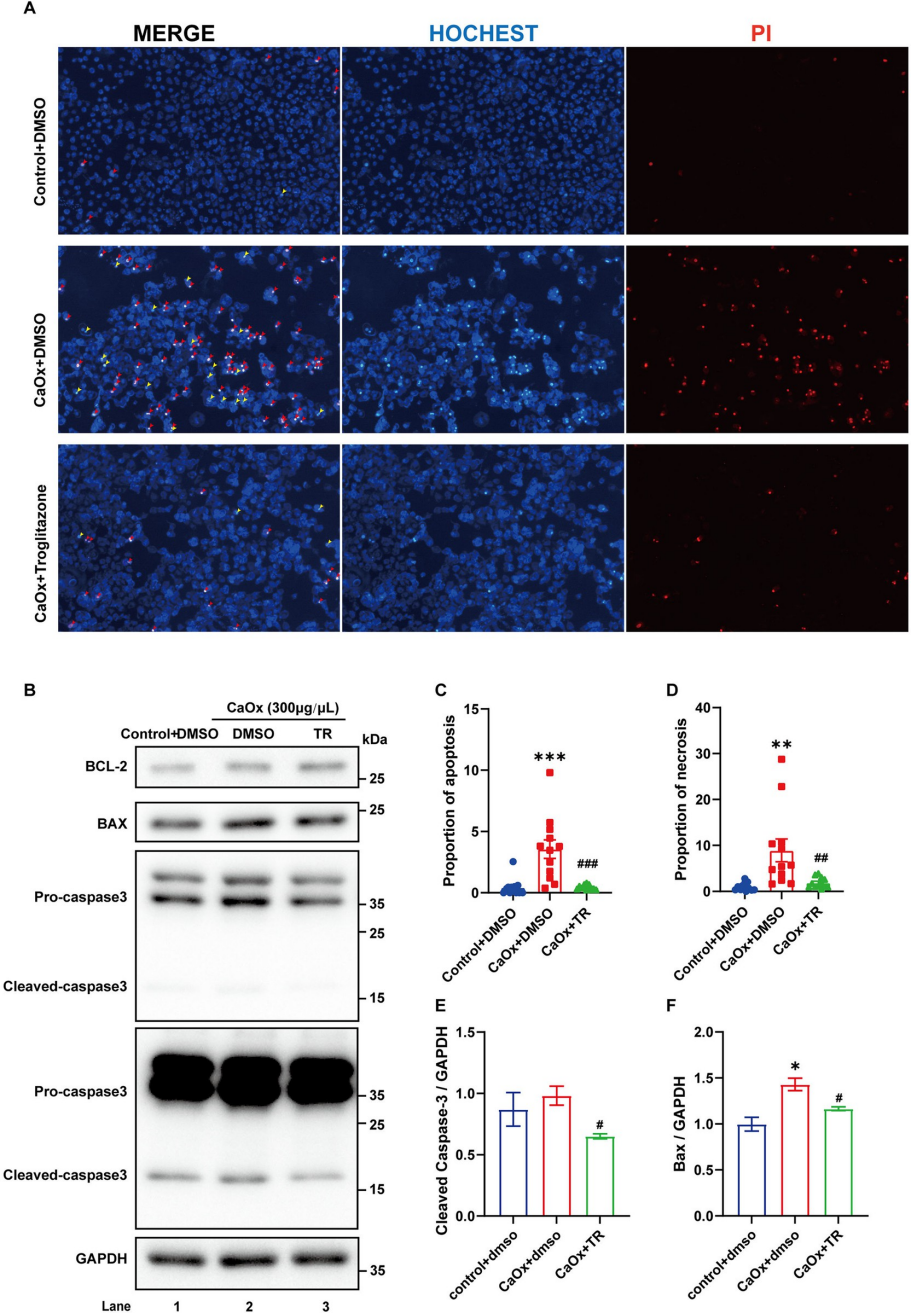
Treatment with a PPARγ agonist improved COM-induced apoptosis in HK2 cells. **(A)** The apoptosis and necrosis rates were evaluated by Hoechst and PI staining (10×). The yellow arrows indicate apoptotic cells, and red arrows indicate necrotic cells. **(B)** Lysates from HK2 cells were analyzed by western blotting with the indicated antibodies. **(C-D)** The proportion of apoptotic to necrotic cells in A, n = 12 fields per group. **(E-F)** The relative protein quantification of B, n = 3 per group. Data are represented as the mean ± SEM; * *P*<0.05, ** *P*<0.01, *** *P*<0.001 *vs*. the control + DMSO group; ^#^ *P*< 0.05, ^##^ *P*< 0.01, ^###^ *P*< 0.001 *vs*. the CaOx + DMSO group.

PPARγ agonists have been used to rescue mitochondrial dysfunction in many studies [24–28] because they can improve mitochondrial biogenesis, dynamics and membrane potential. We pretreated HK2 cells with the PPARγ agonist troglitazone (TR) and found that TR treatment effectively rescued the fragmented mitochondria in COM-treated HK2 cells compared with the CaOx+DMSO group (Fig 4A–4E). At the same time, H2DCF staining of living cells revealed that the elevated ROS levels in COM-treated HK-2 cells were also alleviated by TR treatment (Fig 4F). Consistent with previous findings, western blot and qRT‒PCR analyses both indicated that TR treatment significantly rescued imbalanced mitochondrial dynamic in COM-treated HK-2 cells, with downregulation of DRP1 expression and elevated mRNA levels of PGC1-α and TFAM (Figs 4G–4J, S1A and S1B). As a result, TR treatment significantly improved the apoptosis of COM-treated HK2 cells (Fig 5A–5F). Overall, we believe that calcium oxalate attachment participates in the activation of TEC apoptosis in calcium oxalate nephrolithiasis by affecting mitochondrial dynamic, which could be rescued with PPARγ activation.

### 3.5 PPARγ agonist rescued the renal damage phenotype caused by calcium oxalate crystals

**Fig 6:**
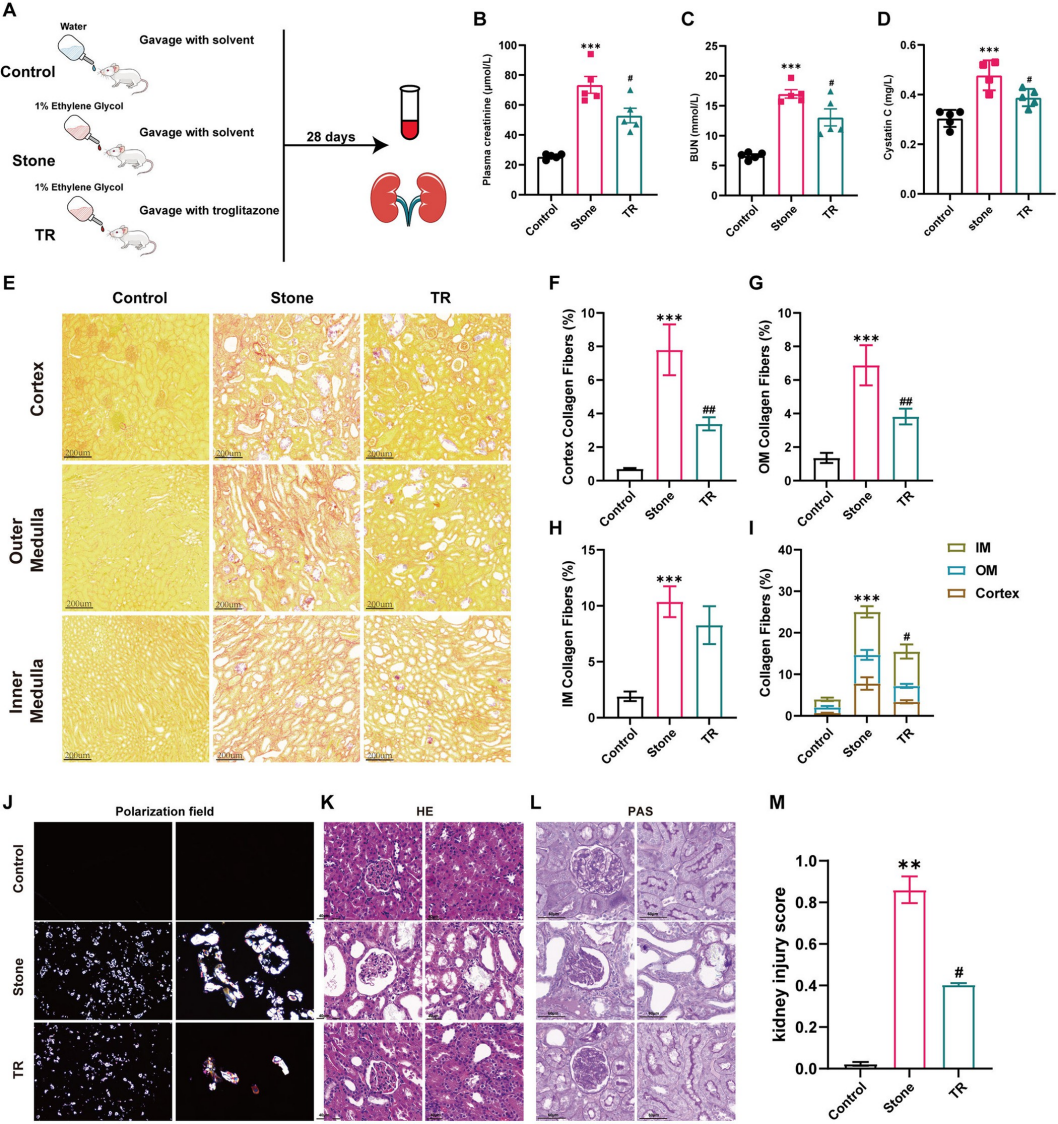
Treatment with a PPARγ agonist improved kidney function and pathological damage induced by calcium oxalate crystals. **(A)** Experimental flowchart for establishment of EG-induced calcium oxalate nephrolithiasis and treatment with troglitazone; **(B)** Plasma creatinine, **(C)** BUN, and **(D)** Cystatin C were measured to assess kidney function in the different groups, n = 5 rats per group; **(E)** The degree of interstitial fibrosis in the renal cortex and outer and inner medulla areas in Sirius Red staining. Scale bar = 200 μm; **(F-I)** The quantification of E, n = 50 fields per group; **(J)** CaOx crystal deposits in the kidney tissues of rats by polarized light optical microphotography (left panel, magnification 10×; right panel, magnification 40×); **(K-L)** Paraffin-embedded sections of renal tissue were stained with H&E (Scale bar = 40 μm) and PAS staining (Scale bar = 60 μm), respectively; **(M)** Kidney injury was scored in the different groups, n = 15 fields per group. Data are represented as the mean ± SEM; * *P*<0.05, ** *P*<0.01, *** *P*<0.001 *vs*. control group; ^#^ *P*< 0.05, ^##^ *P*< 0.01, ^###^ *P*< 0.001 *vs*. stone group.

Given the rescue effects of TR on imbalanced mitochondrial dynamic and apoptosis damage processes *in vitro*, we intended to subsequently validate the effects of PPARγ agonists on renal damage in calcium oxalate nephrolithiasis within the experimental animal model. We administered TR (3 mg/kg) by gavage once a day during model establishment (Fig 6A). Through the analysis of blood samples, we found that TR treatment effectively rescued the reduction in kidney function induced by CaOx, leading to decreased levels of plasma creatinine, urea nitrogen, and cystatin C (Fig 6B–6D, other detailed data can be found in S4 Table in S1 File). At the same time, the expression levels of the proinflammatory cytokines TNF-α, IL-1β, IL-6 and CD68 were significantly decreased in the TR group (S2A–S2D Fig). The Sirius Red staining results also indicated a significant improvement in the fibrosis levels within the renal tissue of the TR group of rats, with a significantly reduced degree of renal tubule dilation compared to the stone group (Fig 6E–6I). Polarized light microscopy revealed a reduced number of calcium oxalate crystals in the renal tissue, and H&E and PAS staining results suggested that TR treatment effectively alleviated the renal damage phenotype induced by CaOx crystals (Fig 6J–6L). Through the quantitative scoring of various pathological indicators of renal damage (Fig 6M), we confirmed that TR treatment indeed improved renal damage in calcium oxalate nephrolithiasis.

### 3.6 PPARγ agonist rescued elevated oxidative stress and TEC apoptosis in the kidney

**Fig 7:**
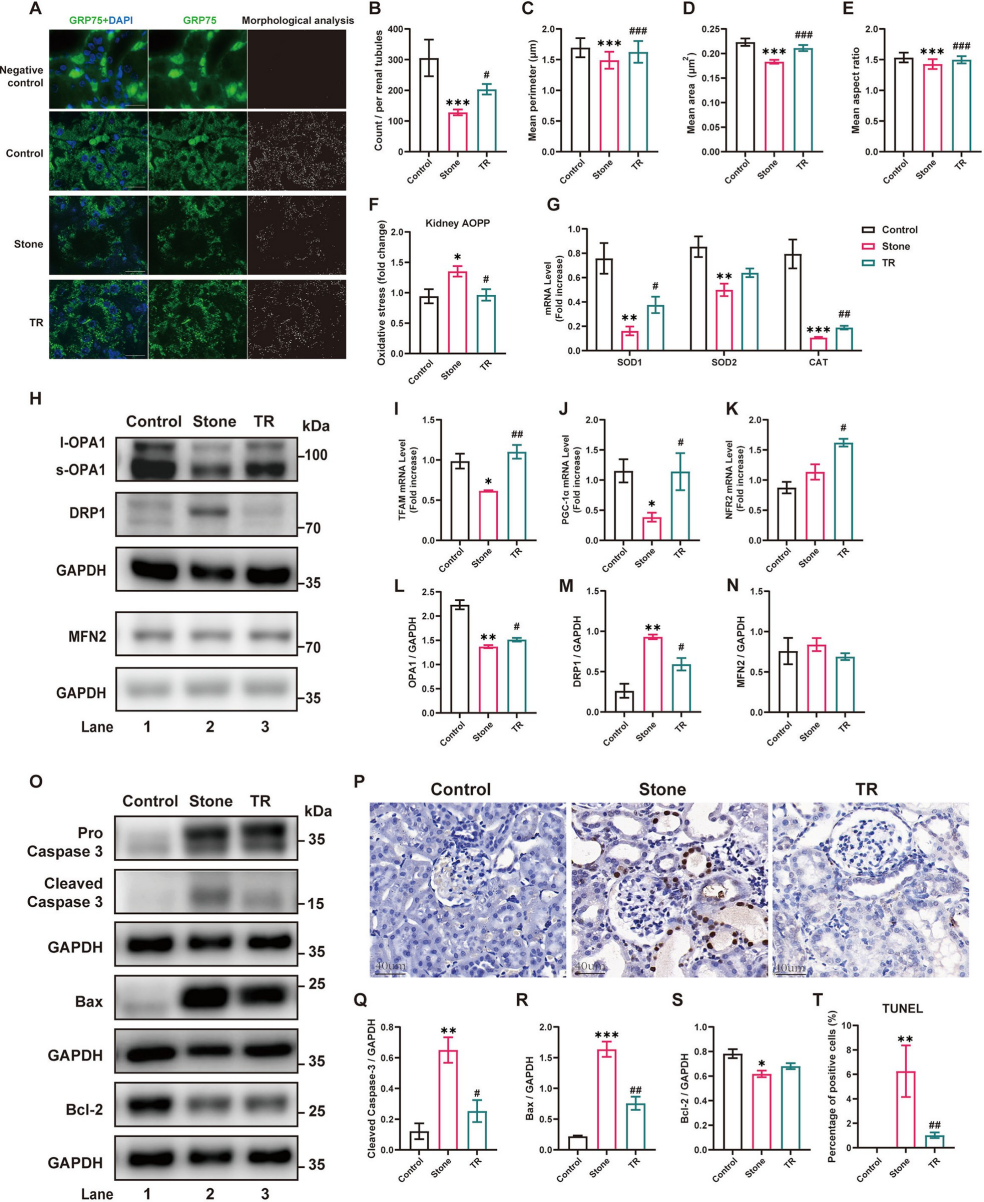
Treatment with a PPARγ agonist improved impaired mitochondrial quality control, oxidative stress, and apoptosis induced by calcium oxalate crystals in the kidney. **(A)** Immunofluorescence staining of the mitochondrial protein GRP75 and schematic representation of mitochondrial morphology in the renal cortex regions of the different groups. Scale bar = 20 μm; **(B-E)** The mean count of mitochondria per renal tubule, mean area (μm^2^), mean perimeter (μm), and mean aspect ratio of mitochondria in different groups were quantified, n = 15 renal tubules per group; **(F)** Oxidative stress levels in renal tissue were measured by the AOPP test; **(G)** Gene expression levels of different renal tissues were determined by RT‒qPCR, n = 5 rats per group; **(H)** Lysates from renal tissue were analyzed by western blotting with indicated antibodies; **(I-K)** Gene expression levels of different renal tissues were determined by RT‒qPCR, n = 5 rats per group; **(L-N)** The relative protein quantification of H, n = 3 rats per group; **(O)** western blotting of renal tissue with indicated antibodies; **(P)** TUNEL staining for apoptotic cells in rat renal tissue. Scale bar = 40 μm; **(Q-S)** The relative protein quantification of O, n = 3 rats per group; **(T)** The proportion of TUNEL-positive cells in P, n = 15 fields per group. Data are represented as the mean ± SEM; * *P*<0.05, ** *P*<0.01, *** *P*<0.001 *vs*. the control group; ^#^ *P*< 0.05, ^##^ *P*< 0.01, ^###^ *P*< 0.001 *vs*. the stone group.

Consistent with the results from the *in vitro* experiments, immunofluorescence staining of the mitochondrial protein GRP75 showed that TR treatment significantly improved the count and morphological changes in mitochondria in TECs of rats kidney compared with the stone group (Fig 7A–7E), which could be attributed to significantly increased OPA1 and decreased DRP1 expression (Fig 7H and 7L–7N). The kidney AOPP test indicated that TR treatment significantly attenuated the elevated oxidative stress in the stone group (Fig 7F). The mRNA expression analysis showed significant downregulation of antioxidant enzymes (SOD1, SOD2, CAT) and genes related to mitochondrial biogenesis (TFAM, PGC1-α and NFR2) in the stone group, which were all upregulated by TR administration (Fig 7G and 7I–7K). Benefiting from the aforementioned mechanism, apoptosis in renal TECs related to calcium oxalate nephrolithiasis showed a significant improvement (Fig 7O–7T).

## 4. Discussion

The deposition of oxalate-induced crystals leading to renal injury is a highly intricate process involving various factors, such as reactive oxygen species (ROS), cellular apoptosis, inflammatory response, and fibrosis [29]. Through our study, we demonstrated that imbalanced mitochondrial dynamics might be a key component in the mechanism of renal dysfunction and showed that treatment with a PPARγ agonist could mitigate oxidative stress-induced injury and TEC apoptosis by improving mitochondrial biogenesis and inhibiting mitochondrial fission.

Mitochondria, as central hubs for metabolism, ion transport, and various macromolecular synthesis pathways, play a critical role in establishing and controlling extensive signaling networks that ensure cellular survival [30]. The survival of renal tubular epithelial cells is vital for maintaining kidney function in patients with kidney stones. Both our research and findings from other teams have shown that calcium oxalate crystals can induce apoptosis in these cells [31]. Mitochondrial damage could lead to apoptosis through the accumulation of ROS, calcium overload, and disruptions in mitochondrial dynamics [32,33]. Our data have proved that the deposition of oxalate-induced crystals leading to imbalanced mitochondrial dynamics in renal tubular epithelial cells, therefore, promoting the normalization of mitochondrial function may be an important strategy for mitigating apoptosis in renal tubular epithelial cells. The kidney is the organ with the highest number of mitochondria inside its cells after the heart, and kidney stone disease has been shown to be associated, either directly or indirectly, with mitochondrial dysfunction [14]. Elevated production of reactive oxygen species (ROS) in mitochondria is closely associated with poor mitochondrial quality control [34,35]. Dynamin-related protein 1 (DRP1) is the sole known protein responsible for mitochondrial fission in mammals. Multiple pieces of evidence have demonstrated that DRP1-induced mitochondrial fragmentation leads to the generation of ROS [33,36]. Previous studies have demonstrated that ROS accumulation can promote damage to renal tubular epithelial cells and crystal deposition [37]. Moreover, ROS act as chemical mediators of inflammation, stimulating the secretion of proinflammatory cytokines and triggering a cascade of inflammatory responses that exacerbate interstitial tissue inflammation [38]. Both oxidative stress and inflammation contribute to the development of fibrosis [39], which is a common and ultimately pathological feature in many chronic inflammatory diseases and can lead to eventual organ dysfunction [40]. Many previous studies have reported the role of elevated ROS in the progression of calcium oxalate nephrolithiasis [7,11,41,42], but there has been relatively little research on the role of mitochondrial dysfunction in this context. Shaoxiong Ming’s study showed oxalate-induced mitochondrial damage in HK2 cells, including lower mitochondrial membrane potential and mitochondrial kinetic imbalance, which was consistent with our data [11]. Jiannan Liu’s research further proved that mtROS from impaired mitochondria are responsible for the inflammatory damage to TECs and the kidney caused by CaOx crystals [43]. Rei Unno’s study primarily focused on the role of autophagy, an essential mechanism for mitochondrial quality control, in kidney stone development [44]. In our study, for the first time, we confirmed the direct damaging effect of calcium oxalate crystals, not oxalate crystals, on mitochondrial dynamic of TECs both *in vitro* and *in vivo* experiments, which should be responsible for the elevated mitochondrial damage, accumulation of reactive oxygen species and TECs apoptosis.

Indeed, previous research has highlighted the immunomodulatory properties of PPARγ, with an emphasis on its anti-inflammatory effects [45–48]. However, in our study, we found that the application of PPAR- agonists have a direct cytoprotective effect on renal TECs, which can alleviate the increased mitochondrial fission in renal tubular epithelial cells induced by stone stimulation, reduce the expression of DRP1, and mitigate TECs apoptosis caused by CaOx crystals stimulation and improve renal function in rats with kidney stones. This is consistent with the research findings of PPARγ in other organs and type of cells. There were two studies that both reported a decrease in PPARγ protein levels in the kidneys of rats with kidney stones [49,50]. However, in our research, the mRNA levels of PPARγ in the kidneys of the model rats were significantly elevated compared to the control group and returned to normal levels after administration of a PPARγ agonist (S2E Fig), which seemed to indicated that the protein levels of PPARγ may regulate its mRNA levels through a negative feedback mechanism to maintain relative stability in its protein levels. Therefore, when kidney stones cause a decrease in PPARγ protein levels, its mRNA levels may increase compensatorily. As the downstream pathways are activated by the PPARγ agonist, the changes in mRNA levels may also be suppressed. Previous research has demonstrated that PPARγ can promote mitochondrial biosynthesis and inhibit mitochondrial fission through PGC1-α upregulation [28,51,52]. PGC1α stimulates the activation of nuclear respiratory factor 2 (NRF2), which in turn activates mitochondrial transcription factor A (TFAM). The activation of the PGC-1α-NRF2-TFAM pathway leads to the synthesis of mitochondrial DNA and proteins, ultimately resulting in the generation of new mitochondria [52,53]. PGC1α can directly regulate the expression of dynamin-related protein 1 (DRP1) by binding to its promoter. PGC1α overexpression can downregulate DRP1 expression, thus alleviating mitochondrial dysfunction and cardiac dysfunction in diabetic mice [28]. In studies related to brain injury, PGC1α activation has been shown to rescue mitochondria by promoting mitochondrial biosynthesis and inhibiting excessive fission, thereby exerting neuroprotective effects [54]. Thus, our study not only discovered the role of imbalanced mitochondrial dynamics in kidney stone-related renal dysfunction but also further elucidated the intrinsic molecular mechanisms by which PPARγ agonist alleviates the progression of kidney stones.

## 5. Conclusion

In conclusion, our results indicated that CaOx crystals could induce significantly imbalanced mitochondrial dynamics in TECs, which should be responsible for elevated ROS, activated apoptosis, fibrosis and inflammation in calcium oxalate nephrolithiasis. Using a PPARγ agonist to restore mitochondrial dynamics could effectively alleviate calcium oxalate-induced renal injury both in vivo and in vitro.

## Supporting information

S1 Fig PPARγ agonist improved COM-induced mitochondrial biogenesis damage in HK2 cells. **(A)** Gene expression levels of PGC-1α in HK2 cells were determined by RT-qPCR, n = 3 per group; **(B)** Gene expression levels of TFAM in HK2 cells were determined by RT-qPCR, n = 3 per group. Data are represented as mean ± SEM; * *P*<0.05 vs control + DMSO group; # *P*< 0.05 vs CaOx + DMSO group.
(TIF)

S2 Fig PPARγ agonist improved the activation of inflammation induced by calcium oxalate in rats. **(A-E)** Gene expression levels of different renal tissues were determined by RT-qPCR, n = 5 rats per group. Data are represented as mean ± SEM; * *P*<0.05, ** *P*<0.01, *** *P*<0.001 vs control group; # *P*< 0.05 vs stone group.
(TIF)

S1 Data (XLSX)

S1 File (DOCX)

S1 Raw images (TIF)
