# Supplementary figures and images for: PPARγ agonist alleviates calcium oxalate nephrolithiasis by regulating mitochondrial dynamics in renal tubular epithelial cell
Source: PLoS One. 2024 Sep 26;19(9):e0310947. doi: 10.1371/journal.pone.0310947 (PMC11426502; doi:10.1371/journal.pone.0310947)

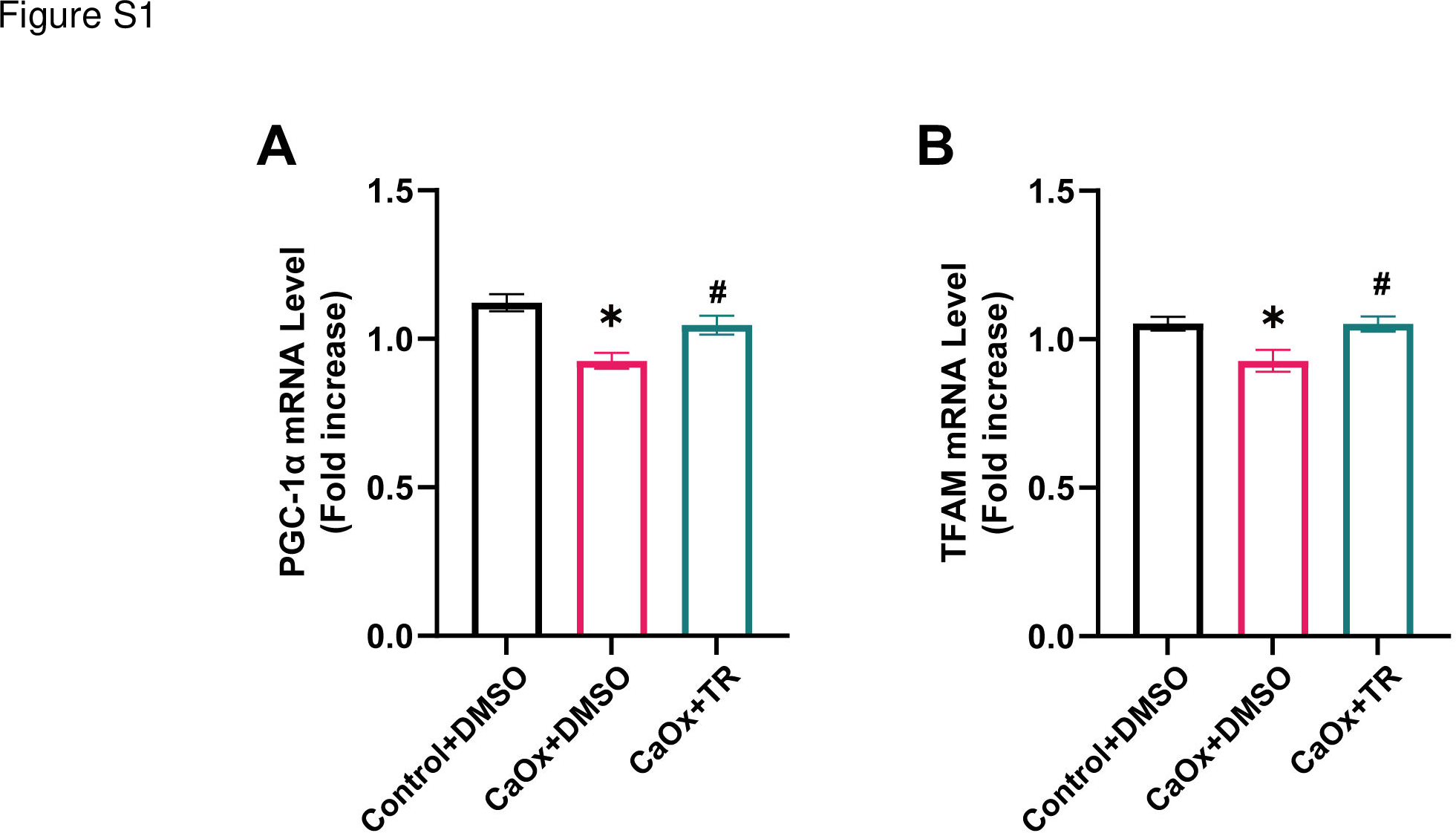

Supplement: S1 Fig — (A) Gene expression levels of PGC-1α in HK2 cells were determined by RT-qPCR, n = 3 per group; (B) Gene expression levels of TFAM in HK2 cells were determined by RT-qPCR, n = 3 per group. Data are represented as mean ± SEM; * P<0.05 vs control + DMSO group; # P< 0.05 vs CaOx + DMSO group. (TIF) [file pone.0310947.s001.tif]

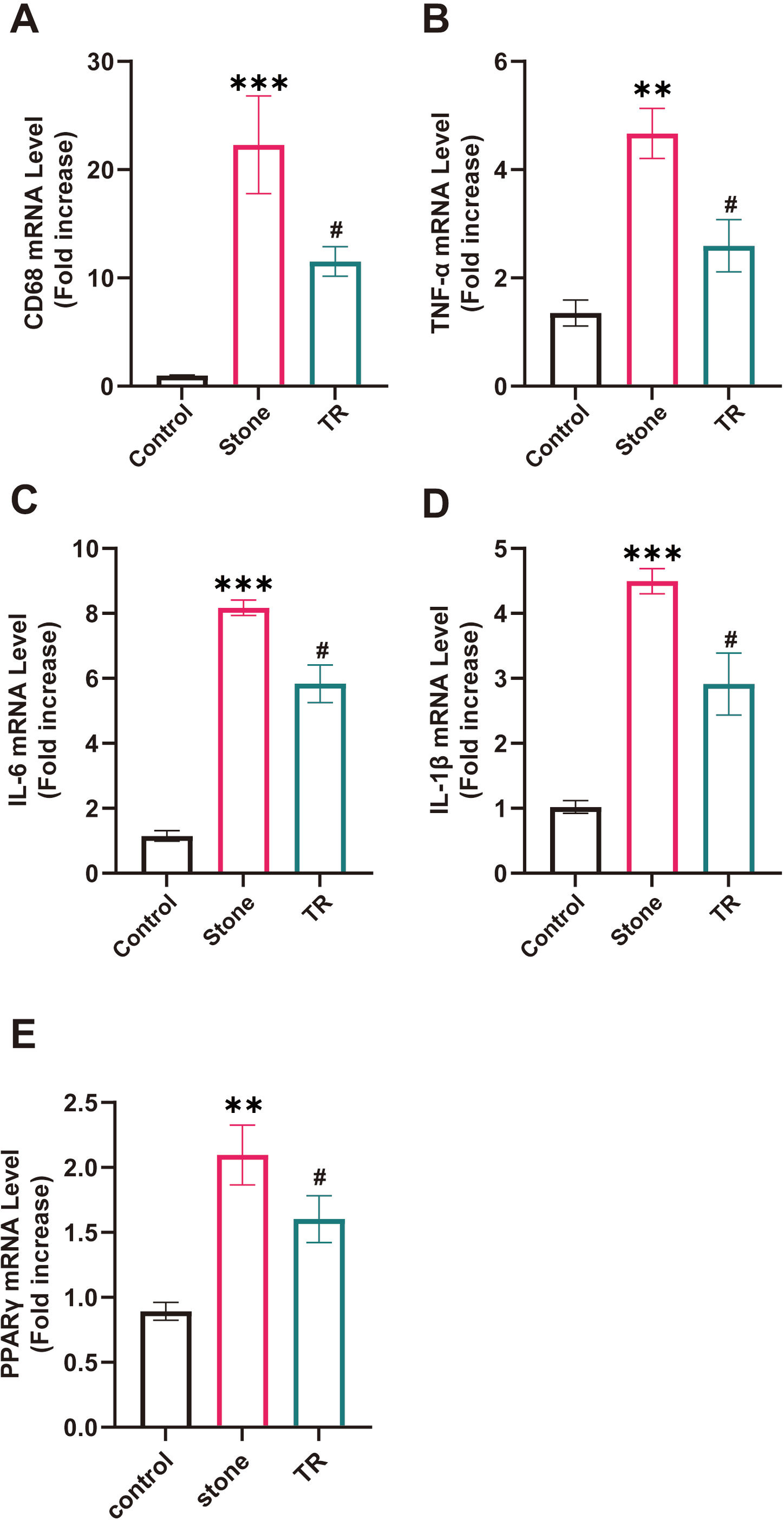

Supplement: S2 Fig — (A-E) Gene expression levels of different renal tissues were determined by RT-qPCR, n = 5 rats per group. Data are represented as mean ± SEM; * P<0.05, ** P<0.01, *** P<0.001 vs control group; # P< 0.05 vs stone group. (TIF) [file pone.0310947.s002.tif]

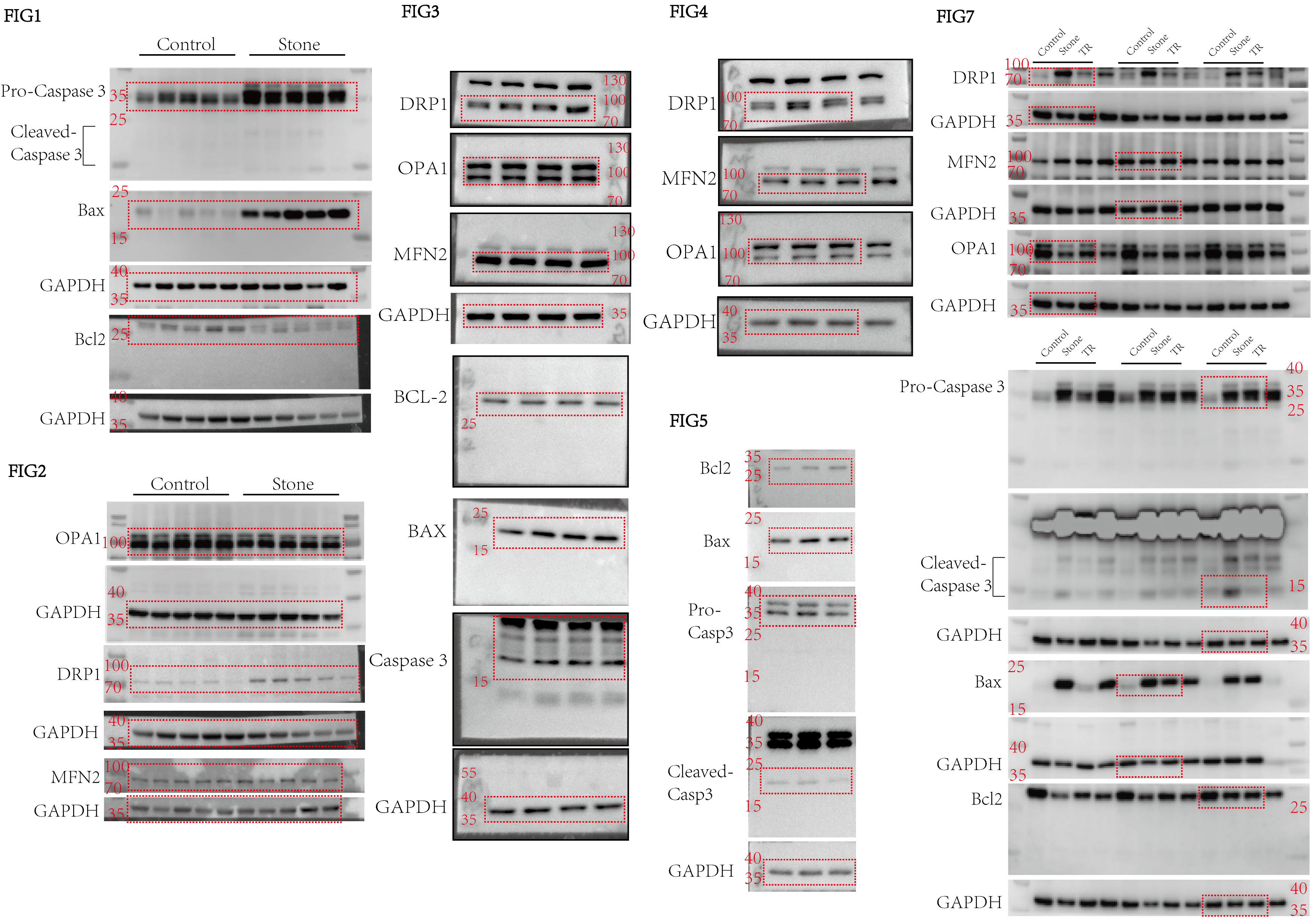

Supplement: S1 Raw images — (TIF) [file pone.0310947.s005.tif]
