# Supplementary material for: PPARγ agonist alleviates calcium oxalate nephrolithiasis by regulating mitochondrial dynamics in renal tubular epithelial cell
Source: PLoS One. 2024 Sep 26;19(9):e0310947. doi: 10.1371/journal.pone.0310947 (PMC11426502; doi:10.1371/journal.pone.0310947)
Supplement: S1 File — (DOCX) [file pone.0310947.s004.docx]

**Supplementary material**

**Supplementary Table S1**

Antibody information.

| **Antibody** | **Type** | **Manufacturer** | **Location** | **Source** | **Catalog #** |
| --- | --- | --- | --- | --- | --- |
| Caspase-3 | monoclonal | Abcam | Cambridge, UK | Rabbit | ab179517 |
| Bax | monoclonal | Abcam | Cambridge, UK | Rabbit | ab32503 |
| Bcl-2 | polyclonal | Abcam | Cambridge, UK | Rabbit | ab196495 |
| MFN2 | monoclonal | CST | Danvers, MA | Rabbit | 9482S |
| OPA1 | monoclonal | CST | Danvers, MA | Rabbit | 67589S |
| DRP1 | monoclonal | Abcam | Cambridge, UK | Rabbit | ab184247 |
| GAPDH | polyclonal | Bioworld | Saint Louis Park, MN | Rabbit | A531 |
| GRP75 | monoclonal | Abcam | Cambridge, UK | Mouse | ab2799 |

**Supplementary Table S2**

Primer sequences of rat used for real-time qPCR analysis

| **Genes** | **Forward (5’-3’)** | **Reverse (5’-3’)** |
| --- | --- | --- |
| Collagen ӀαӀ | TGTTGGTCCTGCTGGCAAGAATG | GTCACCTTGTTCGCCTGTCTCAC |
| Fibronectin-1 | AGGCACAAGGTCCGAGAAGAGG | GGTCAAAGCATGAGTCATCCGTAGG |
| [Vimentin](javascript:;) | TGACATTGAGATCGCCACCT | TCATCGTGGTGCTGAGAAGT |
| α-SMA | GGGCATCCACGAAACCACCT | GAGCCGCCGATCCAGACAGA |
| CD68 | CTCTCTTGCTGCCTCTCATCATTGG | GCTGGTAGGTTGATTGTCGTCTCC |
| Il-1b | AATCTCACAGCAGCATCTCGACAAG | TCCACGGGCAAGACATAGGTAGC |
| Il-6 | AGTTGCCTTCTTGGGACTGATGTTG | GTTGTGGGTGGTATCCTCTGTGAAG |
| TNF-α | CACCACGCTCTTCTGTCTACTGAAC | TGGGCTACGGGCTTGTCACTC |
| SOD1 | GCCGTGTGCGTGCTGAAGG | ACCGCCATGTTTCTTAGAGTGAGG |
| SOD2 | AGCCTCCCTGACCTGCCTTAC | TCGTGGTACTTCTCCTCGGTGAC |
| CAT | GCCGTCCGATTCTCCACAGTC | TGTTTCCCACAAGGTCCCAGTTAC |
| PGC-1α | GACAAGACCAGTGAACTACGGGATG | AAGGAAGAGCAAGAAGGCGACAC |
| NRF2 | ATCTGGAAGTCTTCAGCATGTTACG | TTGTATCTGGCTTCTTGCTCTTGG |
| TFAM | GCAGAAACGCCTAAAGAAGAAAGC | ACTCATCCTTAGCCTCCTGGAAG |
| HO-1 | CCGCCTTCCTGCTCAACATTG | TCTGTGAGGGACTCTGGTCTTTG |
| GAPDH | TGATTCTACCCACGGCAAGTT | TGATGGGTTTCCCATTGATGA |

**Supplementary Table S3**

Primer sequences of human used for real-time qPCR analysis

| **Genes** | **Forward (5’-3’)** | **Reverse (5’-3’)** |
| --- | --- | --- |
| PGC-1α | ACGCACCGAAATTCTCCCTT | TCTGCCTCTCCCTTTGCTTG |
| TFAM | CGCTCCCCCTTCAGTTTTGT | CCACTCCGCCCTATAAGCAT |
| GAPDH | GTCAAGGCTGAGAACGGGAA | AAATGAGCCCCAGCCTTCTC |

**Supplementary Table S4**

Renal function of rats in plasma

| **Groups** | **Control** | **Stone** | **TR** |
| --- | --- | --- | --- |
| [urea](javascript:;) (mmol/l) | 6.6 ± 0.3 | 17.0 ± 0.7^***^ | 13.1 ± 1.4^***,#^ |
| Creatinine (μmol/l) | 25.6 ± 0.7 | 73.5 ± 5.6^***^ | 53.0 ± 4.9^**,#^ |
| K^+^ (mmol/l) | 7.8 ± 0.3 | 6.8 ± 0.3 | 6.8 ± 0.2 |
| Na^+^ (mmol/l) | 141.7 ± 0.8 | 144.3 ± 0.9 | 144.5 ± 0.9 |
| Cl^-^ (mmol/l) | 100.9 ± 0.7 | 100.9 ± 0.3 | 100.6 ± 0.6 |
| Ca^2+^ (mmol/l) | 2.6 ± 0.0 | 2.7 ± 0.0 | 2.7 ± 0.0 |
| P^3+^ (mmol/l) | 2.6 ± 0.1 | 2.7 ± 0.2 | 2.5 ± 0.1 |
| Mg^2+^ (mmol/l) | 1.0 ± 0.0 | 1.1 ± 0.0 | 1.0 ± 0.0 |
| [AG](javascript:;) (mmol/l) | 13.0 ± 1.5 | 20.0 ± 0.6^**^ | 18.8 ± 0.8^**^ |

The number of each group is 5. *p < 0.05, **p < 0.01,***p < 0.001vs control group. ^#^p < 0.05, ^##^p < 0.01, ^###^p < 0.001 vs stone group. AG, anion gap.

**Source Data**

All the data we used for statistics was uploaded to the *Source Data* file*.*

**S1-5,7_Raw_Images**

All original images of western blot in our manuscripts were uploaded to *S1-5,7_Raw_Images* file.
